# Functional analysis of amino acid substitutions within human AGT1 in a cell-based platform to support the diagnosis of primary hyperoxaluria type 1

**DOI:** 10.1016/j.jbc.2025.110494

**Published:** 2025-07-17

**Authors:** Leonardo Gatticchi, Ilaria Bellezza, Gill Rumsby, Michelle Glover, Barbara Cellini

**Affiliations:** 1 Department of Medicine and Surgery, University of Perugia, Perugia, Italy; 2 Kintbury, UK, Formerly Department of Clinical Biochemistry, University College London Hospitals NHS Foundation Trust, London, UK; 3 Rare kidney disease unit, Novo Nordisk Inc, Plainsboro, New Jersey, United States

**Keywords:** Metabolic liver disease, primary hyperoxaluria type 1, alanine: glyoxylate aminotransferase 1, variants of unknown significance, *AGXT*-KO HepG2

## Abstract

Primary hyperoxaluria type 1 (PH1) is caused by the functional deficit of alanine: glyoxylate aminotransferase (AGT1), resulting in a build-up of oxalate. PH1 is diagnosed through the detection of biallelic pathogenic/likely pathogenic variations in the *AGXT* gene. However, the widespread availability of genetic screening has led to an increased identification of novel variants in patients, yet precisely determining their pathogenicity remains a challenge. Since *in silico* tools can give misleading results, functional analyses in disease models are needed to confirm the diagnosis. Here, to help the clinical assessment of *AGXT* variants of uncertain significance (VUS), we implemented a platform based on a cellular model of PH1, made up of HepG2 cells with the endogenous *AGXT* gene knocked out and infected with lentiviral vectors encoding each variant. We generated stable clones expressing the two polymorphic forms of AGT1 (major: AGT1-Ma and minor: AGT1-Mi) as negative controls; five validated pathogenic forms as positive controls; 13 variants classified according to the American College of Medical Genetics and Genomics guidelines as VUS, benign/likely benign, or conflicting. We analyzed each clone for AGT1-specific activity, protein levels, and intracellular localization, as well as for its glyoxylate detoxification ability, as a functional assay. An unbiased global analysis of the data allowed unambiguous clustering of both non-pathogenic and validated pathogenic variants, thus providing information on the possible pathogenicity of each variant. As such, our cell platform represents an important tool that could be applied to a large number of *AGXT* variants to support the diagnosis of PH1.

Primary hyperoxaluria type 1 (PH1; OMIM 259900) is a rare genetic disorder caused by inherited genetic variants on the *AGXT* gene encoding alanine:glyoxylate aminotransferase (AGT1) (1, 2, 3). AGT1 is a liver-specific, vitamin B6–dependent enzyme that catalyzes a transamination reaction to convert glyoxylate into glycine in the peroxisomal matrix (4). Genetic variants in *AGXT* cause a functional deficit in AGT1, which prevents glyoxylate detoxification, thus favoring its cytosolic accumulation and consequent oxidation to oxalate by hepatic lactate dehydrogenase (LDH) (3). The increased endogenous production of oxalate, an end product of human metabolism, generates calcium oxalate stones that deposit in the kidneys and urinary tract, leading to nephrocalcinosis, progressive kidney damage, chronic kidney disease, and kidney failure in most patients (5, 6, 7). In the most severe cases, systemic oxalosis can occur when the increased production of oxalate is compounded by a compromised kidney function, thus causing a life-threatening condition where oxalate deposits in many tissues, including bones, skin, heart, and retina (8).

Based on clinical presentation, the estimated prevalence of PH1 is 1 to 3 per million in Europe and North America (9), with a higher prevalence in developing countries where consanguineous marriages are more common (10); however, whole-exome sequencing data suggest a higher genetic prevalence implying that the disease could be largely undiagnosed (11, 12). The diagnosis of PH1 is established in a proband with hyperoxaluria or hyperoxalemia by genetic testing identifying biallelic pathogenic genetic variants of *AGXT*. The spectrum of PH1-associated genetic variants is highly heterogeneous, with more than 350 genetic variants currently listed in the ClinVar database (13). About 67% of the pathogenic variants currently identified are missense changes involving residues widely spread over the AGT1 structure, in agreement with *in vitro* data indicating that amino acid substitutions can cause the deficit of AGT1 function through a variety of molecular mechanisms, including functional defects (mainly related to compromised catalytic efficiency or coenzyme binding), structural defects (altered folding efficiency or overall stability), and a combination of both (11, 14, 15, 16). The scenario is further complicated by the fact that two polymorphic changes occurring within *AGXT* and generating the so-called “minor allele” (AGT1-Mi) can contribute to the molecular pathogenesis of PH1 by exacerbating the effects of some genetic variants (17, 18, 19, 20). The minor allele, which is found in 15 to 20% of the Caucasian population and in 50% of PH1 patients, is characterized by a 74-bp duplication in intron 1 and two polymorphisms leading to the p.Pro11Leu and the p.Ile340Met amino acid substitutions (21, 22, 23). Despite minor alterations from a catalytic point of view, AGT1-Mi differs from AGT1-Ma by a reduction in thermodynamic and kinetic stability at the protein level. The p.Pro11Leu change is the main cause of AGT1-Mi destabilization, by enhancing its aggregation and degradation propensities (17, 24), as well as by generating a weak mitochondrial targeting sequence (MTS) at the N-terminus of AGT1 (2, 22). The MTS is ineffective on AGT1-Mi, but when co-inherited with genetic variants affecting folding kinetics, it can lead to the mistargeting of the protein to mitochondria, where AGT1 is metabolically ineffective (25, 26).

The ease of access to genetic testing and population studies has greatly improved PH1 diagnosis in the last years, but they have also increased the number of new variants identified. Although the consequences of some missense changes have been defined, the impact of many novel variants on AGT1 expression and function still remains unknown, thus complicating clinical assessment. Prediction software, segregation studies, and prevalence analyses of the normal population and affected families can provide some indications; however, for most novel variants, classification as variants of unknown significance (VUS) remains.

The American College of Medical Genetics and Genomics (ACMG) suggests classifying newly identified sequence variants as pathogenic, likely pathogenic, likely benign, or benign, and supporting genetic testing with *in vitro* or *in vivo* functional tests of the gene product (27, 28). Our group has recently implemented a novel PH1 cellular model based on HepG2 cells—a human cell line with conserved glyoxylate/oxalate metabolism derived from hepatocellular carcinoma—knocked out for the *AGXT* gene (*AGXT*-KO HepG2) using CRISPR/Cas9 technology (29). *AGXT*-KO HepG2 cells represent a suitable model to reproduce the pathogenicity of PH1-associated variants as they mimic hepatocyte metabolism and protein homeostasis (29).

In this work, we exploited this model to investigate the functional properties of a panel of *AGXT* VUS obtained from the gnomAD (the Genome Aggregation Database, https://gnomad.broadinstitute.org/), ClinVar (https://www.ncbi.nlm.nih.gov/clinvar/), and ACMG (https://www.acmg.net/) databases, and associated with either the major or minor allele. Each variant was stably expressed in *AGXT*-KO HepG2 cells using lentiviral vectors, and the cells were analyzed for AGT1 expression, specific activity, subcellular localization, and oxalate excretion/release with and without glycolate challenge. A global analysis of the data allowed us to establish a platform for reliable VUS classification.

## Results and discussion

### Expression of *AGXT* VUS in a cellular disease model

**Figure 1:**
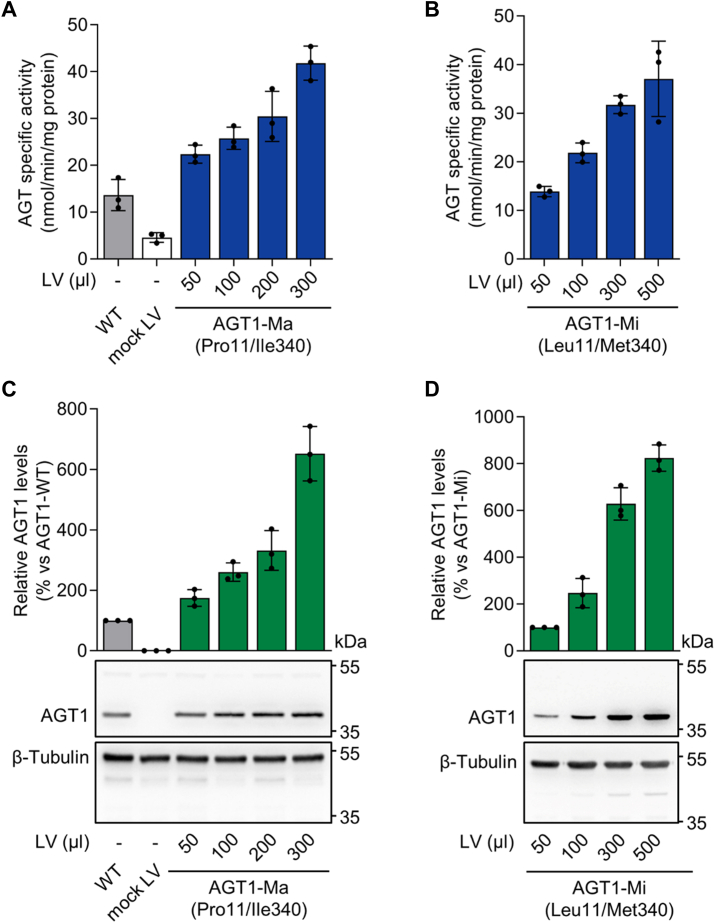
**Lentivirus-mediated expression of human alanine:glyoxylate aminotransferase (AGT1) polymorphic forms in an *in vitro* model of primary hyperoxaluria type 1 (PH1).***AGXT*-KO HepG2 cells were transduced with different amounts of lentiviral vectors carrying *AGXT* cDNA encoding for AGT1 polymorphic forms (50–500 μl of supernatants containing lentiviral particles). Specific AGT transaminase activity was assessed in transduced *AGXT*-KO HepG2 cells with (*A*) AGT1 major (AGT1-Ma) and (*B*) AGT1 minor (AGT1-Mi) polymorphic forms by a standard spectrophotometric assay. AGT1 protein levels were measured by immunoblotting in total cell lysates. Representative protein blots from three independent experiments are shown. Graph represents the densitometric analysis of AGT1 protein levels as the relative expression of AGT1-Ma normalized to β-tubulin levels, in respect to (*C*) wild-type (WT) HepG2 cells or (*D*) AGT1-Mi (50) expressing cells. Results represent mean ± standard deviation (SD) of n = 3 independent transductions. LV, lentivirus.

Immortalized cell lines represent a valuable tool to study the impact of inherited missense genetic variants on the overall fitness of a target protein and can provide insights into the pathogenicity of VUS (30). Broadly used cells, such as Chinese hamster ovary (CHO) cells in PH1, ensure easy management and high expression levels but do not always mimic the physiological environment of the target protein (22, 31, 32, 33, 34, 35). To overcome this problem and better mimic the hepatic microenvironment, we used a new PH1 *in vitro* model based on *AGXT*-KO HepG2 cells (29). Vectors suitable for lentiviral infection encoding AGT1-Ma and AGT1-Mi were constructed. Infection of *AGXT*-KO HepG2 cells with increasing amounts of lentiviral particles encoding AGT1-Ma (Fig. 1, panels A and C) or AGT1-Mi (Fig. 1, panels *B* and *D*) led to stable cell clones displaying a corresponding increase in transaminase-specific activity and AGT1 expression levels (Fig. 1). Note that *AGXT*-KO HepG2 cells display residual transaminase activity due to enzymatic activity other than AGT1, and that the specific activity of cells expressing AGT1-Mi is about 83% of that of cells expressing AGT1-Ma, in line with previous data obtained in different cellular models (29). Since our study goal was to provide quantitative evidence for the impact of missense genetic variants at the protein level, the experimental platform was set up to obtain comparable amounts of transduced DNA for each cell clone (Fig. S1), ensuring AGT1 levels and specific activity are similar to those of wild-type cells (Fig. 1).

Overall, our data demonstrate that this system provides a cost-effective approach for heterologous expression of AGT1 in hepatic-origin cells, enabling the evaluation of missense genetic variants affecting glyoxylate/oxalate metabolism. Therefore, we used this platform to analyze the properties of a group of 18 *AGXT* variants involving residues spanning the entire protein sequence. The group comprises five validated pathogenic variants associated with either the major (p.Gly41Arg, p.Ser158Leu, and p.Asp183Asn) or minor (p.Gly170Arg and p.Phe152Ile) alleles, serving as positive controls. Additionally, we examined five variants of uncertain significance (VUS), four with conflicting interpretations, three classified as benign or likely benign, and one unclassified variant (Tables 1 and 2) summarizes the preliminary evaluation of each variant using available *in silico* tools whose readout is mainly based on sequence and structure conservation. This information clearly shows that, although the five validated genetic variants are considered damaging by most software, it is difficult to obtain definitive results without further clinical and biochemical analyses. It must also be considered that *in silico* analyses do not consider the allelic background of a genetic variant, nor the possible effect of a genetic variant on AGT1 subcellular localization, two aspects that can be crucial in influencing the penetrance and severity of PH1 (12, 21, 36).

**Table 1:** List of *AGXT* (NM_000030.3) missense variants analyzed

Nucleotide variant	Protein consequence	ClinVar classification	ACMG classification (after manual curation)	References
c.82C>T	p.Pro28Ser	VUS	PM2 PM3 PP3 BP2 VUS	(71)
c.121G>A	p.Gly41Arg	P (on both alleles)	PS3 PM2 PM3 PP3 PP4 PP5 Pathogenic	(19, 22, 29, 40, 41, 72)
c.352C>T	p.Arg118Cys	VUS	PS3 PM2PM5 PP3 PP4 Pathogenic	
c.385G>C	p.Asp129His	VUS	PM2 PP3 BS3 VUS	(18, 58)
c.454T>A	p.Phe152Ile	P	PS3 PM2 PM3 PP3 PP4 PP5 Pathogenic	(26, 58, 73, 74, 75, 76)
c.473C > T	p.Ser158Leu	P/LP	PM2 PM3 PP3 PP4 PP5 Likely pathogenic	(11, 24, 46, 47, 48, 77)
c.508G > A	p.Gly170Arg	P/LP	PS3 PS4 PM2 PM3 PP1 PP3 PP5 Pathogenic	(58, 73, 74, 78, 79)
c.547G>A	p.Asp183Asn	LP	PVS1 PS3 PM2 PM3 Pathogenic	(46, 49, 50)
c.557C>T	p.Ala186Val	B/LB	BS1 BS2 BP2 BP6 benign	(58, 80, 81)
c.590G>A	p.Arg197Gln	B/LB	BS1 BS2 BP2 BP6 benign	(58, 80, 81)
c.743C>T	p.Ala248Val	Conflicting	PM2 PP3 VUS	
c.822G>C	p.Glu274Asp	VUS	PS3 PS5 PM2 PP1 PP3 PP4 Pathogenic	
c.836T>C	p.Ile279Thr	B/LB	On minor allele PS3 PM2 PM3 PP3 Likely pathogenic On major allele (57) PM2 PP3 BS3 BP2 BP6 Likely benign	(58)
c.865C>T	p.Arg289Cys	VUS	PM2 BS3 BP2 likely benign	(59, 82)
c.866G>A	p.Arg289His	Conflicting	PM2 PM3 BP2 BP4 Likely benign	(83)
c.942G>A (splice site)	p.Pro314Pro	VUS	n.a.	n.a.
c.949C>T	p.Arg317Trp	Conflicting	PM2 PM3 PP3 PP5 Likely Pathogenic	(11, 84)
c.1093G>T	p.Gly365Cys	n.a.	PS3 PS5 PM2 PM3 PP3 Pathogenic	(41)

ACMG, American College of Medical Genetics and Genomics; AGT1, alanine:glyoxylate aminotransferase 1; B, benign.

LB, likely benign; LP, likely pathogenic; n.a., not available; P, pathogenic; VUS, variants of unknown significance.

**Table 2:** Prediction of *AGXT* variants pathogenicity by using *in silico* tools

Amino acid substitution	PolyPhen-2	SIFT	CADD	REVEL	MetaLR	Mutation assessor	Mutation taster
p.Pro28Ser	Probably damaging (0.999)	Deleterious (0)	Likely benign (23)	Likely disease causing (0.743)	Damaging (0.969)	High (0.966)	Deleterious (0.999)
p.Gly41Arg	Possibly damaging (0.765)	Deleterious (0.01)	Likely benign (20)	Likely disease causing (0.751)	Damaging (0.877)	Medium (0.651)	Deleterious (0.999)
p.Arg118Cys	Probably damaging (1)	Deleterious (0)	Likely benign (26)	Likely disease causing (0.808)	Damaging (0.838)	High (0.978)	Deleterious (0.999)
p.Asp129His	Possibly damaging (0.522)	Deleterious (0.01)	Likely benign (25)	Likely disease causing (0.687)	Damaging (0.791)	Medium (0.753)	Benign (0)
p.Phe152Ile	Probably damaging (0.953)	Deleterious (0)	Likely benign (25)	Likely disease causing (0.869)	Damaging (0.852)	Medium (0.609)	Deleterious (0.999)
p.Ser158Leu	Probably damaging (0.968)	Deleterious (0.01)	Likely deleterious (31)	n.a.	Damaging (0.792)	Medium (0.914)	Deleterious (0.999)
p.Gly170Arg	Probably damaging (0.998)	Deleterious (0)	Likely benign (24)	Likely disease causing (0.908)	Damaging (0.883)	High (0.95)	Deleterious (0.999)
p.Asp183Asn	Probably damaging (1)	Deleterious (0)	Likely benign (27)	n.a.	Damaging (0.992)	High (0.985)	Deleterious (0.999)
p.Ala186Val	Benign (0.184)	Tolerated (0.05)	Likely benign (17)	Likely disease causing (0.613)	Tolerated (0.151)	Medium (0.879)	Benign (0)
p.Arg197Gln	Benign (0)	Tolerated (1)	Likely benign (19)	Likely benign (0.364)	Tolerated (0.023)	Medium (0.009)	Benign (0)
p.Ala248Val	Possibly damaging (0.626)	Deleterious (0)	Likely benign (25)	Likely disease causing (0.651)	Damaging (0.76)	Medium (0.781)	Deleterious (0.998)
p.Glu274Asp	Probably damaging (0.964)	Deleterious (0)	Likely benign (29)	Likely disease causing (0.84)	Damaging (0.831)	High (0.977)	Deleterious (0.999)
p.Ile279Thr	Possibly damaging (0.839)	Deleterious (0)	Likely benign (22)	Likely disease causing (0.551)	Damaging (0.685)	Medium (0.842)	Benign (0)
p.Arg289Cys	Possibly damaging (0.675)	Deleterious (0.01)	Likely benign (25)	Likely disease causing (0.703)	Damaging (0.79)	Medium (0.805)	Deleterious (0.998)
p.Arg289His	Benign (0.009)	Tolerated (0.11)	Likely benign (17)	Likely disease causing (0.563)	Damaging (0.656)	Medium (0.621)	Benign (0)
p.Arg317Trp	Probably damaging (1)	Deleterious (0)	Likely benign (28)	Likely disease causing (0.809)	Damaging (0.897)	Medium (0.838)	Deleterious (0.999)
p.Gly365Cys	Probably damaging (1)	n.a.	n.a.	n.a.	n.a.	n.a.	Deleterious (0.999)

Pathogenicity interpretation and scoring for each *in silico* tool used: PolyPhen-2 (<0.49 benign, 0.5–0.9 possibly damaging, > 0.9 probably damaging); SIFT (<0.05 deleterious, > 0.05–1.0 tolerated); CADD (<30 likely benign, > 30 likely deleterious); REVEL (<0.5 likely benign, > 0.5 likely disease causing); MetaLR (<0.5 tolerated, > 0.5–one damaging); Mutation assessor (damaging score between 0–1, neutral, low, medium, or high); Mutation taster (0 benign, 1 deleterious); n.a., not available.

### Generation and characterization of *AGXT* variants on the background of the major allele

Selected clones of *AGXT*-KO HepG2 cells infected with lentiviral vectors expressing each variant were checked to ensure that each one displayed a similar level of exogenous *AGXT* inserted in the genomic DNA, to guarantee that the observed changes were due to protein-related alterations and not to different infection efficiency (Fig. S2*A*).

**Figure 2:**
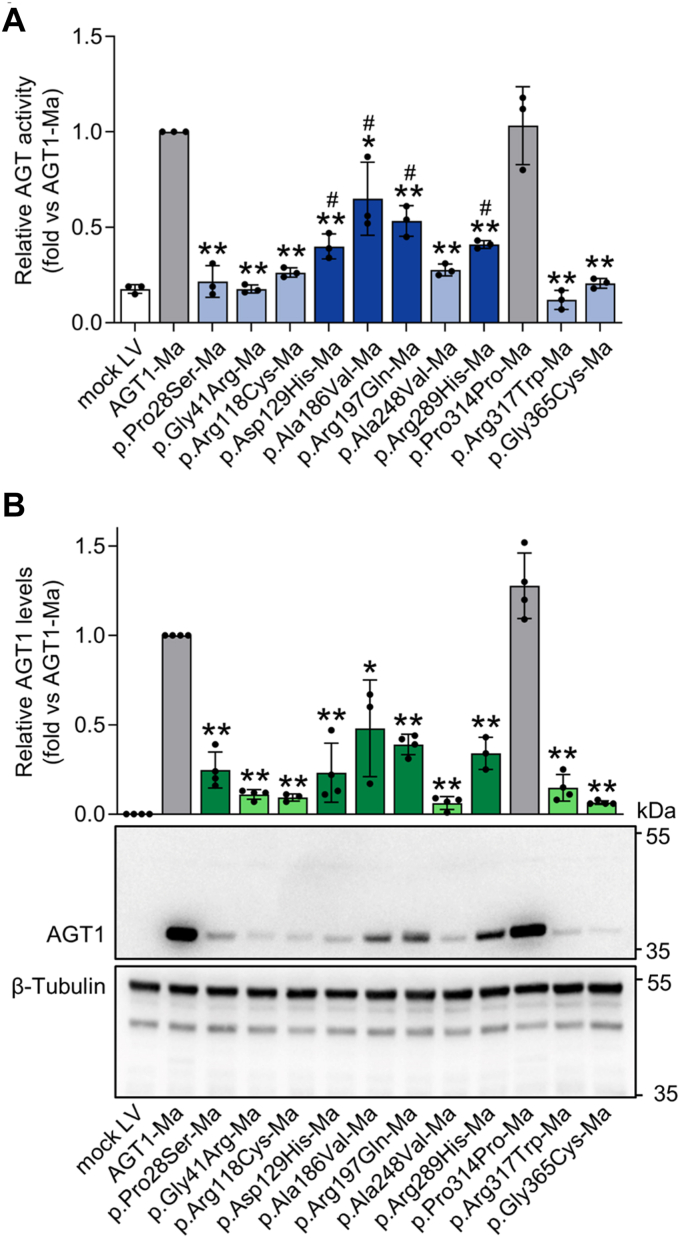
**Expression and activity of *AGXT* variants of uncertain significance (VUS) associated with alanine:glyoxylate aminotransferase major (AGT1-Ma) background**. *AGXT*-KO HepG2 clones expressing variants associated with AGT1-Ma polymorphic form were obtained by lentiviral vectors infections. *A*, AGT specific transaminase activity was assessed in transduced cells by a spectrophotometric assay (n = 3–5). The mean value of specific AGT transaminase activity in cells expressing AGT1-Ma was assumed as 1. All other transaminase activity values were related to those of AGT1-Ma. *B*, representative image of *AGXT*-KO HepG2 cell clones expressing different *AGXT* VUS associated with AGT1-Ma. β-Tubulin was used as loading control. Graph represents the mean mutant AGT1 protein levels (fold) normalized to β-tubulin levels and relative to AGT1-Ma (n = 3–4). Data are expressed as mean ± standard deviation (SD). ∗*p* < 0.05 and ∗∗*p* < 0.01 *versus* AGT1-Ma; #*p* < 0.05 *versus* mock LV. LV, lentivirus.

All the genetic variants expressed on AGT1-Ma background, except the Pro314 synonymous change, significantly reduced AGT1 levels and activity (Fig. 2). Absence of protein-related alterations on the c.942G>A nucleotide change (Pro314Pro) validated the system as the negative control. Nonetheless, since the point genetic variant resides in a region of the primary transcript involved in splicing, its consequences on the final mRNA product, if any, remain to be determined. Among the genetic variants giving rise to statistically significant alterations, different behaviors can be observed for each parameter. Regarding activity, some genetic variants gave rise to a moderate reduction in specific activity (approx. 40−60% of the value of AGT1-Ma, bright blue in Fig. 2*A*), while others led to a strong effect (light blue in Fig. 2*A*), with specific activity values not significantly different from those of the empty vector (*e.g.*, mock LV). As for protein levels, a group of variants displayed a moderate reduction of protein levels (bright green in Fig. 2*B*), while another group displayed protein levels less than 25% relative to AGT1-Ma (light green in Fig. 2*B*). All produced proteins were responsive to an antibody against Pro11, thus confirming the allelic background (Fig. S2*B*).

A combined analysis of the two parameters allows us to draw the following conclusions: a first group of genetic variants (p.Asp129His, p.Ala186Val, p.Arg197Gln, p.Arg289His) has a moderate effect on both activity and protein levels. As for Asp129, no remarkable effects of its genetic variation to histidine have been found upon expression in yeast and COS-7 cells (18). However, Asp129 is located in a peripheral loop region of the large domain of AGT1 that shows increased structural flexibility and is prone to disorder. Genetic variants in this region have been found to reduce protein levels and specific activity in mammalian cells (17), in line with our results. Notably, the loop region containing Asp129 displays an increased flexibility in AGT1-Mi, suggesting that this genetic variant could give rise to more pronounced effects on the minor allele background. Ala186 is part of a random-coil region located near the active site, where other pathogenic variants have been found, such as p.Ser187Phe, which strongly affects coenzyme binding and catalysis (37, 38). It can be speculated that the observed mild effects could be caused by the conservative Ala-to-Val substitution. A similar conclusion can be drawn for the Arg197 genetic variant, in line with previous data in both purified AGT1 and CHO cells. Indeed, the substitution of Arg197 with a glutamine residue disrupts a salt bridge of the large domain and forms a new hydrogen bond with the N-terminus (39). Finally, no specific molecular studies have been performed on the role of Arg289. The finding that this residue is located in a surface region of AGT1 could explain why the substitution to histidine does not strongly reduce protein intracellular fitness. This agrees with genetic studies indicating that the c.866G > A (p.Arg289His) is a common polymorphism in the African American population (11).

A second group of genetic variants (p.Gly41Arg, p.Arg118Cys, p.Ala248Val, p.Arg317Trp, p.Gly365Cys) gave rise to strong reductions in AGT1 levels and activity. Regarding p.Gly41Arg, our data are consistent with the established pathogenicity of this genetic variant, which thus represents a positive control, as well as with cellular data indicating that the Gly41Arg substitution substantially reduces AGT1 folding/stability (22, 40). As for the p.Gly365Cys, a preliminary *in vitro* study by our group showed that the amino acid substitution interferes with the active site conformation, leading to an altered binding of pyridoxal 5′-phosphate (PLP), which, by shifting the equilibrium toward the apo-form of the enzyme, reduces intracellular stability and activity (41). Therefore, these data further corroborate the pathogenicity of the p.Gly365Cys genetic variant. On the other hand, the functional data presented herein provides the first molecular characterization of the p.Arg118Cys, p.Ala248Val, and p.Arg317Trp genetic variants. Arg118 represents a key residue for AGT1 dimerization, as shown by the destabilizing effects of its mutation to alanine on dimer stability (42), thus suggesting that its mutation to cysteine could promote AGT1 monomerization. Since AGT1 monomerization in turn favors aggregation and intracellular degradation, it is plausible that the observed effects of the Arg118 mutation on protein levels and activity observed in HepG2 cells are directly due to dimer destabilization (43). Ala248 is part of a hydrophobic cluster along with Ile244 and Leu247. The finding that the mutation of Ile244 to Thr is a PH1-causing genetic variant common in the Canary Islands (44) and leads to AGT1 aggregation corroborates the idea that genetic variants in this region could interfere with proper folding. Arg317 is part of the small domain of AGT1, and it does not directly interact with PLP. Although a precise definition of the defect of the p.Arg317Trp mutation would require studies on the purified protein, it can be hypothesized that the substitution of a charged residue with a bulky hydrophobic amino acid could lead to a conformational change that might increase either AGT1 degradation or aggregation propensity, rather than causing a catalytic defect, in line with the observed reduction in protein levels and specific activity.

Of note, the p.Pro28Ser genetic variant led to markedly reduced AGT1-specific activity despite a moderate effect on protein levels. Since Pro28 is part of a loop region located at the entrance of the active site (residues 24–32) connected with the N-terminus (40), the p.Pro28Ser change could compromise substrate binding to affect catalysis (45), thus causing a defect that is both structural and functional. This hypothesis could explain the stronger effect displayed by the mutation on specific activity rather than on protein levels. Indeed, it must be considered that Western blot quantitative analyses inform protein levels under denaturing conditions, which include both folded and unfolded fractions and it may differ from the levels of catalytically active protein.

**Figure 3:**
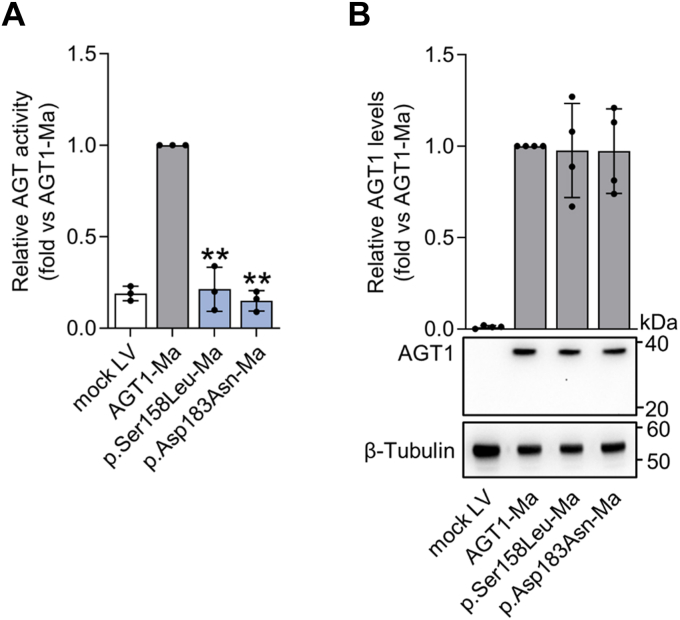
**Expression and activity of catalytically defective *AGXT* variants associated with alanine:glyoxylate aminotransferase major (AGT1-Ma) background**. *AGXT*-KO HepG2 clones expressing variants associated with AGT1-Ma polymorphic form were obtained by lentiviral vectors infections. *A*, AGT-specific transaminase activity was assessed in transduced cells by a spectrophotometric assay (n = 3). The mean value of specific AGT transaminase activity in cells expressing AGT1-Ma was assumed as 1. All other transaminase activity values were related to those of AGT1-Ma. *B*, representative image of *AGXT*-KO HepG2 cell clones expressing catalytically defective *AGXT* variants associated with AGT1-Ma. β-Tubulin was used as loading control. Graph represents the mean mutant AGT1 protein levels (fold) normalized to β-tubulin levels and relative to AGT1-Ma (n = 4). Data are expressed as mean ± standard deviation (SD). ∗∗*p* < 0.01 vs AGT1-Ma. LV, lentivirus.

Following this path, a combined analysis of the data reveals a general correlation between the reduction in specific activity and in protein levels (Fig. S3), indicating that the decrease in activity is due to a decrease in the amount of protein produced. Therefore, we tested if the created platform could be also useful to recognize the pathogenicity of mutations giving rise to catalytic defects, without affecting AGT1 folding. We constructed vectors encoding the p.Ser158Leu and the p.Asp183Asn mutants and analyzed their protein levels and specific activity upon expression in *AGXT*-KO HepG2 cells (Figs. 3 and S3). In line with the expected outcomes, both p.Ser158Leu and the p.Asp183Asn expressing clones displayed transaminase specific activity similar to mock cells, whereas the protein levels were unaffected (Fig. 3, *A* and *B*). Results agree with previous data showing a catalytic impairment due to the substitutions of residues Ser158 and Asp183, which are both involved in PLP binding within the AGT1 active site (24, 46, 47, 48, 49, 50).

### Generation and characterization of *AGXT* variants on minor allele background

**Figure 4:**
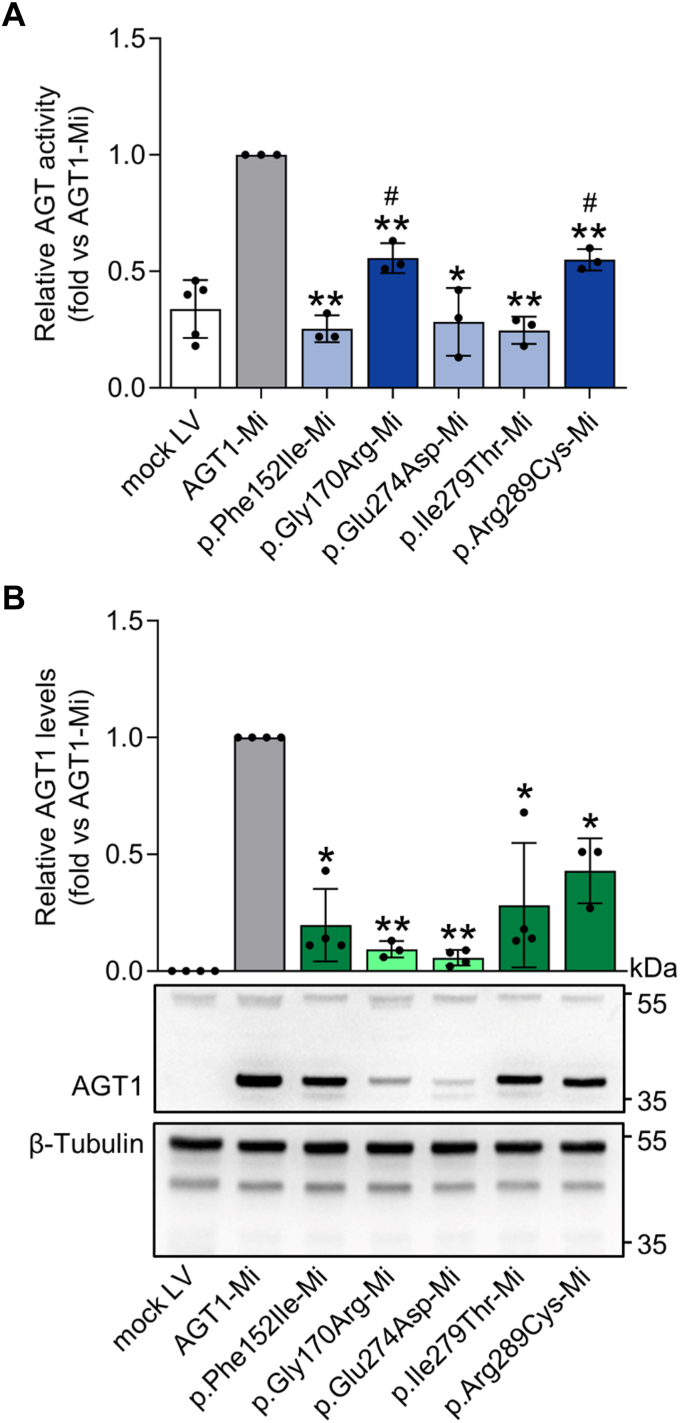
**Expression and activity of *AGXT* variants of unknown significance (VUS) associated with alanine:glyoxylate aminotransferase minor (AGT1-Mi) background**. *AGXT*-KO HepG2 clones expressing variants associated with AGT1-Mi were obtained by transduction with lentiviral vectors. *A*, AGT-specific transaminase activity was assessed in transduced *AGXT*-KO HepG2 cells by a spectrophotometric assay (n = 3). The mean value of specific AGT1 transaminase activity in cells expressing AGT1-Mi was assumed as 1. All other transaminase activity values were related to those of AGT1-Mi. *B*, representative image of *AGXT*-KO HepG2 cell clones expressing different *AGXT* VUS associated with AGT1-Mi. β-Tubulin was used as loading control. Graph on panel B represents the mean mutant AGT1 protein levels (fold) normalized to β-tubulin levels and relative to AGT1-Mi (n = 3–6). Data are expressed as mean ± standard deviation (SD). ∗*p* < 0.05 and ∗∗*p* < 0.01 *versus* AGT1-Mi; #*p* < 0.05 *versus* mock LV. LV, lentivirus.

**Figure 5:**
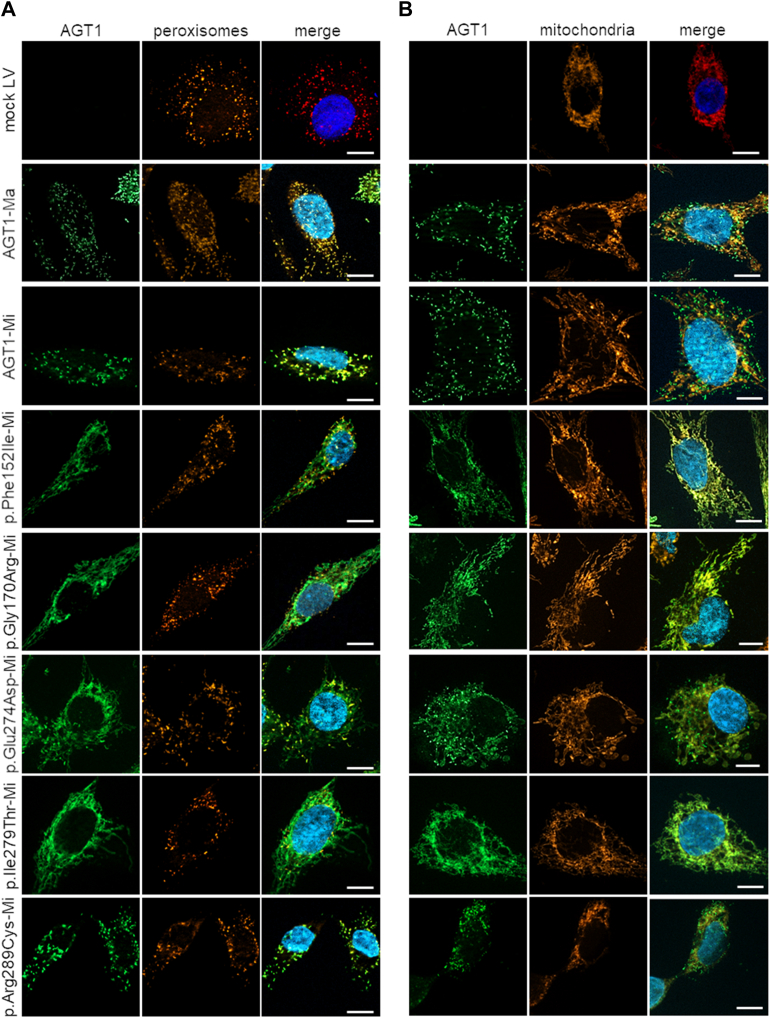
**Subcellular localization of *AGXT* variants associated with the alanine:glyoxylate aminotransferase (AGT1) minor allele (AGT1-Mi).** Intracellular localization of AGT1 mutants was investigated by immunofluorescence in *AGXT*-KO HepG2 cells expressing *AGXT* variants associated with AGT1-Mi. Cells were fixed and stained with antibodies against AGT1 (*green*), and (*A*) peroxisome or (*B*) mitochondria (*red*). Peroxisomes were stained with anti-catalase antibody while mitochondria were stained with MitoTracker. Nuclei were stained with Hoechst 33342 (*blue*). Magnification 63×, scale bar 10 μm.

Using the same strategy employed to analyze variants on the background of the major allele, we selected and validated clones of *AGXT*-KO HepG2 cells infected with lentiviral vectors expressing variants on the minor background of AGT1 (AGT1-Mi), and we compared their specific activity and protein levels with the corresponding control (*e.g.*, AGT1-Mi) (Figs. 4 and S4). The p.Phe152Ile, p.Glu274Asp, and p.Ile279Thr expressing clones displayed specific activity levels not significantly different from those of cells infected with the empty vector (light blue, Fig. 4*A*), thus leading to a strong effect, whereas the p.Gly170Arg and p.Arg289Cys preserved a residual activity equal to 50% of AGT1-Mi activity (bright blue, Fig. 4*A*). Moreover, all genetic variants significantly reduced protein levels relative to AGT1-Mi, although the effect was more pronounced (<25% of the control, light green, Fig. 4*B*) for the p.Gly170Arg, and p.Glu274Asp variants, whereas a moderate effect was observed for the p.Phe152Ile, p.Ile279Thr, and p.Arg289Cys variants (bright green, Fig. 4*B*). Given that the polymorphic p.Pro11Leu amino acid change generates a putative MTS on AGT1 (2, 22), the subcellular localization of each species was also evaluated (Fig. 5). Clear peroxisomal localization was seen for AGT1-Ma and AGT1-Mi. Similarly, the p.Arg289Cys genetic variant, although on the AGT-Mi background, did not significantly affect AGT1 import to peroxisomes. On the other hand, the p.Phe152Ile, p.Gly170Arg, and p.Ile279Thr genetic variants redirected most AGT1 to mitochondria, whereas the p.Glu274Asp genetic variant gave rise to a mixed peroxisomal/mitochondrial localization.

Overall, the enzymatic phenotype of the p.Phe152Ile and p.Gly170Arg genetic variants is in line with their known pathogenicity, which is due to structural changes that reduce AGT1 kinetic and thermodynamic stability, and that promote its mitochondrial mistargeting by delaying the folding process and the interaction with molecular chaperones (22, 33, 42, 51, 52, 53, 54, 55, 56). The finding that the residual-specific activity is lower than that expected from the residual whole protein expression indicates that part of the protein could be present in an inactive form in the cell, in line with previous data indicating that p.Phe152Ile with AGT1-Mi undergoes a time-dependent inactivation at physiological temperature (52). The p.Glu274Asp genetic variant does not completely redirect AGT1 to mitochondria but has a strong effect on protein fitness, in line with previous data obtained using a yeast model and purified proteins (18, 47). Indeed, Glu274 is part of a conserved region of AGT1, and its mutation to aspartate synergizes with the minor allele polymorphism by altering the tertiary structure of holo-AGT1 and compromising PLP binding, thus reducing enzymatic activity and protein stability. Ile279 is not conserved on the AGT1 sequence, and its mutation to threonine has been classified as benign based on data obtained on the purified variant on the background of the major allele, which showed unaltered enzymatic activity, in line with the position of the residue far from the active site (57). In our experimental setting, the genetic variant is associated with the p.Pro11Leu and p.Ile340Met polymorphic changes (AGT1-Mi), and the two amino acid substitutions synergize in reducing the overall intracellular fitness of the protein, as confirmed by the reduction in specific activity and protein levels. This finding was also confirmed in a liver biopsy from an individual showing the importance of knowing the haplotype in this disease (58). Finally, the possible effects of the p.Arg289Cys genetic variant have been only examined in yeast-based assays, in which AGT1 stability was unaffected (47). However, a patient bearing the p.Arg289Cys genetic variant in heterozygosity with the p.Leu298Pro showed early stone events leading to nephrolithiasis and nephrocalcinosis (59). Our results show that the mutation does not affect AGT1 localization, but significantly reduces specific activity and protein levels, although the effects seem mild compared with those of the other genetic variants. Since Arg289 is located on the AGT1 surface, it can be speculated that the substitution to Cys could lead to protein aggregation due to disulfide crosslinking of the mutant protein, especially in an oxidative environment such as that of peroxisomes (60).

### Functional assay of *AGXT* variants on glyoxylate detoxification

**Figure 6:**
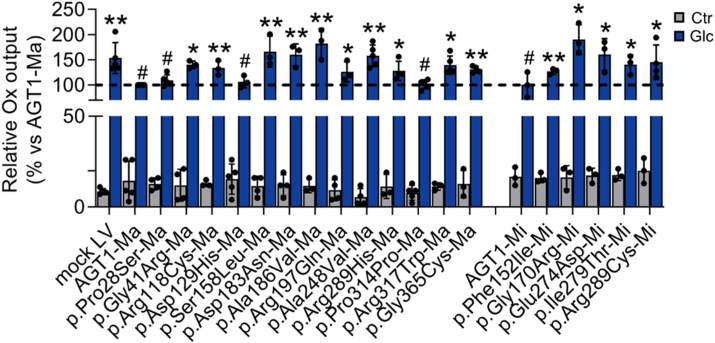
**Relative oxalate release in medium of cells expressing *AGXT* variants**. *AGXT*-KO HepG2 cells stably expressing *AGXT* variants were transiently transfected with pcDNA3.1 vector carrying human *HAO1* cDNA encoding for glycolate oxidase (GO). After 24 h of transfection, cells were incubated in the presence or absence (Ctr, untreated cells) of glycolate (Glc, 10 mM) for another 24 h. Cell media were collected, and oxalate concentration was measured using a spectrophotometric assay. Oxalate levels in medium of *AGXT*-KO HepG2 cells expressing *AGXT* variants of unknown significance (VUS) were associated with the corresponding AGT1 polymorphic form (n = 3–4). Data are expressed as mean ± standard deviation (SD). ∗*p* < 0.05 and ∗∗*p* < 0.01 vs. AGT1-Ma. #*p* < 0.05 vs. mock LV. Ctr, control (untreated cells); Glc, glycolate; LV, lentivirus; Ox, oxalate.

To test whether the analyzed genetic variants affect the ability of AGT1 to perform the detoxification of glyoxylate, we set up a secondary assay aimed at quantifying oxalate production upon cell challenge with glycolate, a glyoxylate precursor. As GO, the enzyme responsible for the glycolate-to-glyoxylate conversion inside peroxisomes, is expressed at low levels in HepG2 cells, we first performed a transient transfection of human *HAO1* (encoding GO) in *AGXT*-KO HepG2 cell clones and then challenged cells with an excess of glycolate (29). As shown in Fig. S5, the increase in glycolate oxidase (GO) expression upon transfection is paralleled by the increase in the amount of oxalate released in the medium upon 10 mM glycolate challenge. When GO transfection and glycolate challenge were performed in clones of *AGXT*-KO HepG2 cells expressing each variant under study (Fig. 6), it was observed that: (i) cells expressing AGT1-Ma and AGT1-Mi show a low amount of oxalate released, in line with their physiological ability to convert glyoxylate to glycine; (ii) cells expressing the p.Pro28Ser, p.Asp129His, and p.Pro314Pro variants release an amount of oxalate not different from that of control cells; (iii) cells expressing the p.Phe152Ile, p.Arg197Gln, and p.Arg289His variants show a moderate increase in oxalate release, while (iv) cells expressing the p.Gly41Arg, p.Arg118Cys, p.Ser158Leu, p.Gly170Arg, p.Asp183Asn, p.Ala186Val, p.Ala248Val, p.Glu274Asp, Ile279Thr, p.Arg289Cys, p. Arg317Trp, and p.Gly365Cys genetic variants release oxalate amounts similar to those of *AGXT*-KO HepG2 cells. In the case of the p.Asp129His, p.Arg197Gln, p.Arg289His, and p.Pro314Pro genetic variants, these results agree with the moderate or absent effects of the genetic variants on enzyme activity and protein levels in the cells (Fig. 2). As for the p.Pro28Ser genetic variant, it is unclear how a variant displaying a strongly reduced specific activity could perform glyoxylate detoxification. It can be speculated that the local glyoxylate concentration in peroxisomes could overcome the functional defect of the variant by favoring substrate binding. Of note, except for p.Ala186Val, and the two known catalytically defective mutants p.Ser158Leu and p.Asp183Asn, all species showing strongly increased oxalate production also display a strong reduction in specific activity and/or protein levels, including the three known pathogenic variants of AGT1. Although it cannot be excluded that GO transfection could have an impact on *AGXT* expression *via* an unknown gene regulatory mechanism, this could be considered unlikely in our cell system because the expression of each genetic variant is under the control of the constitutive CMV promoter. Nevertheless, it must be considered that the experimental setting in which we analyzed oxalate production includes a substrate challenge that on the one hand allows insights into the functionality of each species, but on the other hand creates an environment far from equilibrium, thus influencing the relationship between steady-state levels of functional protein and glyoxylate detoxification ability. In the case of variants associated with the minor allele, the possible effect of aberrant localization must be considered.

### *AGXT* VUS classification

**Figure 7:**
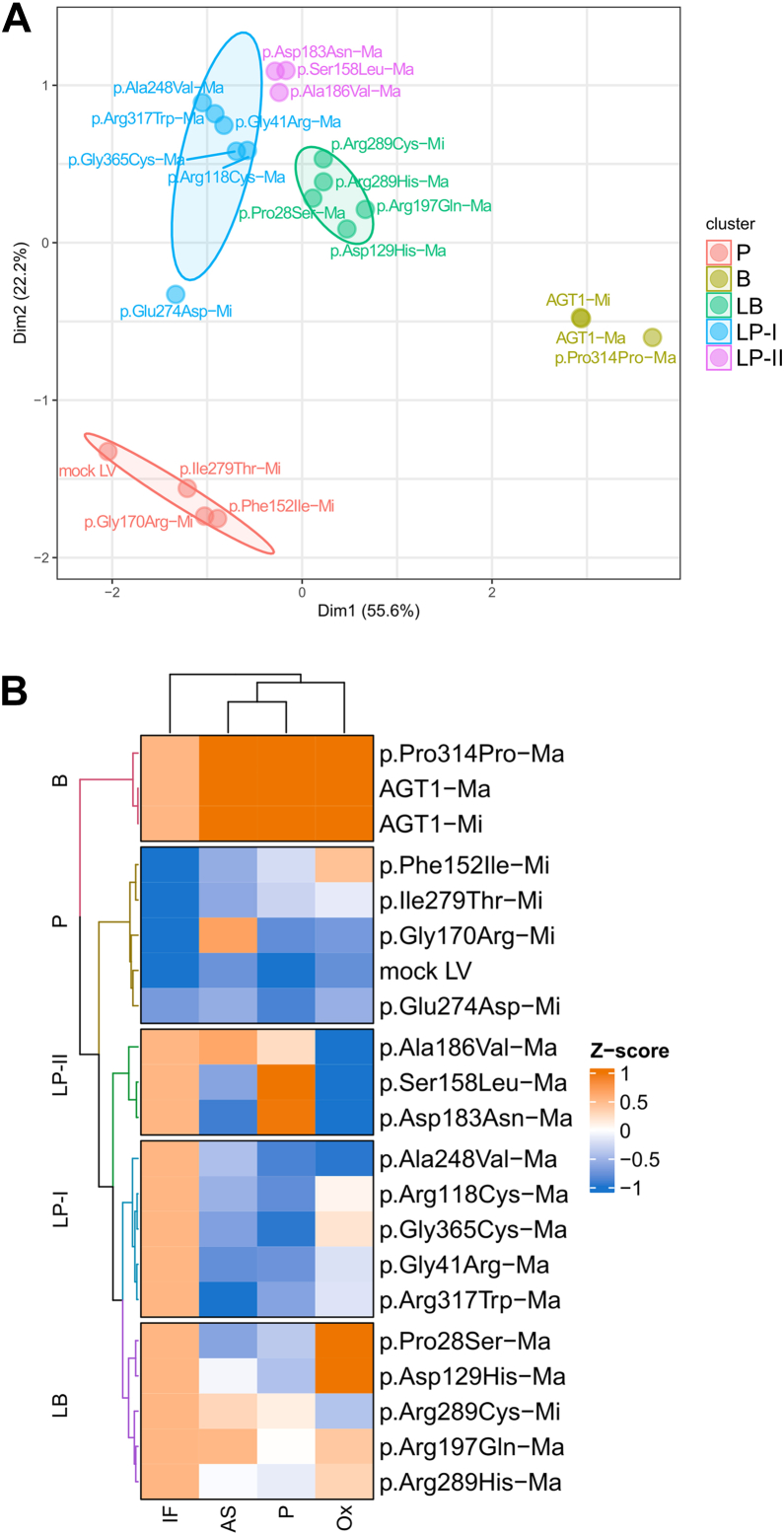
**Principal component analysis (PCA) for *AGXT* variants of unknown significance (VUS) pathogenicity evaluation**. *A*, PCA plot, each variant is represented as a distinct dot in the plot of the first two components, based on its residual specific activity, relative protein levels, glyoxylate detoxification ability, and intracellular localization. Alanine:glyoxylate aminotransferase (AGT1) polymorphic forms (AGT1 major [AGT1-Ma]/AGT1 minor [AGT1-Mi]) and benign variants (*light green*) are clustered on the center right of the graph, whereas likely benign (*green*), likely pathogenic (I, *light blue*; II *purple*), and pathogenic (*pink*) are clustered on the *center top*, *top left*, and *bottom left* of the graph, respectively. Over 77% of the variance in *AGXT* VUS profile data sets was described by the first two components: dim1 (55.6%) and dim2 (22.2%). B, benign; LB, likely benign; LP, likely pathogenic; P, pathogenic. *B*, Hierarchical clustering of *AGXT* VUS visualized by heat map. Heat map was constructed to investigate the relationships and clustering patterns among different *AGXT* VUS based on the assessed variables. Both rows and columns were clustered using correlation distance and average linkage. AS, specific activity; IF, immunofluorescence; LV, lentivirus; Ox, glyoxylate detoxifying ability; P, protein level.

To globally analyze the obtained data according to all available parameters (*i.e.*, residual protein level, specific activity, glycolate detoxification, and protein localization), we applied a principal component analysis (PCA) multivariate statistical approach with hierarchical clustering to group samples, as a tool to obtain insights into their possible pathogenic effects. As shown in Figure 7, *A* and *B*, both PCA plot and heat map clearly show *AGXT* VUS clustering that allows VUS classification between deleterious and tolerated variant groups. The analysis revealed that the variants under study can be grouped into five different clusters, with the two principal components contributing to 78% of the variance in the data set. (i) Cluster 1 comprises benign variants (B), *i.e*., the two polymorphic forms of AGT1 and, as expected, the Pro314 synonymous substitution; (ii) Cluster 2 includes p.Pro28Ser-Ma, p.Asp129His-Ma, p.Arg197Gln-Ma, p.Arg289Cys-Mi, and p.Arg289His-Ma genetic variants, which can be classified as likely benign (LB); (iii-iv) Cluster 3 and 4 comprises likely pathogenic (LP) genetic variants that are additionally subdivided into two distinct clusters based on the type of molecular alteration introduced in the AGT1 molecule. Specifically, a first group (LP-I) gathers genetic variants mainly affecting AGT1 folding and protein stability (*i.e*., p.Arg118Cys-Ma, p.Ala248Val-Ma, p.Glu274Asp-Mi, p.Arg317Trp-Ma, and p.Gly365Cys-Ma); notably, the pathogenic p.Gly41Arg-Ma genetic variant is also included in this group, possibly because its effects are milder if the genetic variant is on the background of the major allele (61). On the other hand, a second cluster of LP variants (LP-II) includes the p.Ala186Val-Ma variant along with the p.Ser158Leu-Ma, p.Asp183Asn-Ma known catalytically defective mutants, possibly underlying a similar impact of the Ala186Val substitution at a molecular level; (v) Cluster 5 includes *AGXT* knock-out (mock LV) cells along the two most common pathogenic genetic variants (p.Phe152Ile-Mi and p.Gly170Arg-Mi), which thus represent a good positive control, as well as the p.Ile279Thr-Mi genetic variants, which can be classified as pathogenic based on the cellular studies.

Overall, our analysis allows us to provide a reliable classification cell-based system for those *AGXT* VUS that previously had conflicting interpretations obtained from *in silico* (Table 2) or clinical/experimental evidence (Table 1). Specifically, of the 12 *AGXT* VUS tested, 8 result in a novel classification (benign: p.Pro28Ser-Ma, p.Asp129His-Ma, p.Arg289Cys-Mi, and p.Arg289His-Ma; disease causing: p.Arg118Cys-Ma, p.Ala248Val-Ma, p.Glu274Asp-Mi, and p.Arg317Trp-Ma), 2 have a confirmed classification (benign: p.Arg197Gln-Ma; disease causing: p.Gly365Cys-Ma), and 2 were reclassified as pathogenic (p.Ala186Val-Ma and p.Ile279Thr-Mi).

## Conclusion

In this work, we aimed at constructing an *in vitro* platform to investigate the pathogenicity of missense genetic variants on the *AGXT* gene associated with either major or minor polymorphic forms of AGT1. We used HepG2 cells knocked out for the endogenous *AGXT* and infected with lentiviral vectors encoding mutant AGT1, a cost-effective approach for the expression of missense variants and semi-quantitative evaluation of their intracellular fitness. Laboratory geneticists have long been challenged by providing meaningful genetic reports to health care providers when minimal variant data are available. Functional studies can elucidate the mechanism and extent to which missense genetic variants affect protein fitness (*i.e.*, its overall function and/or stability) and thus provide insights into their contribution to disease susceptibility. Strong *in vitro* or *in vivo* evidence of pathogenicity or benign impact per ACMG/Association for Molecular Pathology criteria enables reclassification of VUS (27, 62) provided that validated, reproducible, and robust assays are used (27, 63). The platform described herein, through the combined analysis of data on the molecular/cellular consequences of *AGXT* genetic variants on AGT1, allowed us to attribute the genetic variants under analysis to five clusters, which could reliably correspond to benign, likely benign, likely pathogenic, and pathogenic, as confirmed by internal positive and negative controls.

We must acknowledge that our experimental design is not suitable for testing the effects of genetic variants affecting the promoter region or the primary transcript maturation process. Nonetheless, it provides a useful platform for exploring the effect of amino acid substitutions on protein global fitness, thus representing a tool to predict the pathogenicity of newly identified genetic variants and, paired with clinical data, to better understand their potential to manifest PH1. In this regard, it must be mentioned that our PCA analysis included two parameters that are intertwined, *i.e.*, variant residual specific activity and the corresponding protein levels. Indeed, in many cases, the reduction in specific activity is due to intracellular instability, thus leading to reduced protein levels. Nevertheless, the separation of the two parameters can allow a more accurate distinction among mutations giving rise a parallel reduction of specific activity and protein levels and those causing a pure catalytic defect, where a strong reduction in specific activity is observed in the absence of a concomitant reduction in protein levels. Thus, the usage of opportunely selected known pathogenic mutants as references confers to our platform the prediction power needed to discriminate benign and pathogenic variants, and possibly predict, among the pathogenic ones, variants producing purely catalytic defects from the inducing protein misfolding.

However, we must consider that our study has been carried out in a small set of genetic variants, and that an extension of the study to a larger group and number of measured variables could strengthen the accuracy of the analysis and allow a more robust classification of known and newly identified genetic variants. Moreover, it must be considered that even genetic variants classified as likely benign in our platform could give rise to a pathogenic effect in the presence of a different allelic background and/or in compound heterozygosity with another genetic variant that gives rise to a synergic alteration of the structural/functional properties of AGT1. Nonetheless, our platform could support the diagnosis of PH1 starting from a comprehensive analysis that considers not only the clinical features of patients and evaluates their genetic data, but also the effects of each identified genetic variant in standardized functional assays. This could be an important premise to establish genotype/phenotype correlations, as well as to perform preclinical tests of therapeutic efficacy of pyridoxine therapy and putative new drugs.

## Experimental procedures

### Materials

PLP, L-alanine, sodium glyoxylate, and rabbit muscle LDH were purchased from Merck Life Science. Oligonucleotides for site-directed mutagenesis and genome editing screening were obtained from Bio-Fab Research srl. The lentiviral transfer vector carrying codon-optimized *AGXT* cDNA (lenti-hCMV-*AGXT*_BC132819-OP-IRES-puro) was purchased from transOMIC Technologies (Huntsville) and used to obtain VUS encoding transfer vectors by site-directed mutagenesis (see, Generation and characterization of AGXT variants on minor allele background). Packaging (pCMVR8.74) and envelope (pCMV-VSV-G) plasmids were kindly provided by Prof. Leonardo Salviati (University of Padova). Rabbit polyclonal anti-AGT1 and guinea pig anti–peroxisomal catalase antibodies were gifted by Prof. C. J. Danpure (University College London) (64). Rabbit polyclonal anti-GO (PA5-62006) antibody was obtained from Thermo Fisher Scientific. Mouse monoclonal anti–β-tubulin (66,240-1-lg) was obtained from Proteintech. Anti-rabbit (1706515) and anti-mouse (A90-116P) horseradish peroxidase (HRP) secondary antibodies were purchased from Bio-Rad Laboratories and Bethyl Laboratories, respectively. All other chemicals were of analytical grade.

### Cell culture

HepG2 cells were a gift from Prof. Brunetti-Pierri (Telethon Institute of Genetics and Medicine). *AGXT-*KO HepG2 cells were generated as previously described (29) and maintained in high-glucose Dulbecco’s modified Eagle’s Medium (DMEM) containing 2 mM L-glutamine and 1 mM sodium pyruvate (D6429; Merck Life Science) and supplemented with 10% fetal bovine serum (FBS; Gibco, Thermo Fisher Scientific), 100 U/ml penicillin, 0.1 mg/ml streptomycin, and 50 μg/ml gentamicin (G1397; Merck Life Science).

HEK293FT cells were a gift from Prof. Leonardo Salviati (University of Padova). They were grown and used for lentiviral vector preparation as previously described (65) but with slight modifications to the medium composition as follows: high-glucose DMEM containing 2 mM L-glutamine and 1 mM sodium pyruvate and supplemented with 10% non–heat-inactivated FBS, 4 mM L-glutamine (6 mM final concentration), 0.1 mM non-essential amino acids (NEAA; M7145; Merck Life Science), 100 U/ml penicillin, 0.1 mg/ml streptomycin, 50 μg/ml gentamicin, and 500 μg/ml of geneticin (G418 Sulfate; Thermo Fisher Scientific). All cells were maintained in a 5% CO_2_-/water-saturated incubator at 37 °C. All the cell lines and clones used were mycoplasma-negative by PCR (forward primer: 5′GGGAGCAAACAGGATTAGATACCCT3 ′; reverse primer: 5′TGCACCATCTGTCACTCTGTTAACCTC3′).

### *In silico* prediction of variants pathogenicity

Prediction of variant pathogenicity based on conservation score or amino acid substitutions impact assessment, when available, were retrieved from Ensembl database (https://www.ensembl.org/index.html, last accession on February 6, 2025), or by using the following *in silico* tools: PolyPhen-2 (Polymorphism Phenotyping v2; http://genetics.bwh.harvard.edu/pph2/index.shtml), SIFT (Sorting Intolerant from tolerant, http://sift.jcvi.org/), CADD (Combined Annotation Dependent Depletion, https://cadd.bihealth.org/, REVEL (Rare Exome Variant Ensemble Learner, https://sites.google.com/site/revelgenomics/), MetaLR, Mutation Assessor (http://mutationassessor.org/r3/), and Mutation Taster (https://www.mutationtaster.org/).

### Site-directed mutagenesis

The lentiviral transfer vector encoding the human AGT1 major polymorphic form (AGT1-Ma) was first used to prepare the vector encoding the AGT1 minor polymorphic form (AGT1-Mi), with two sequential rounds of site-directed mutagenesis to introduce the two amino acid substitutions (*i.e.*, p.Pro11Leu and p.Ile340Met) (41). Next, selected VUS and known pathogenic *AGXT* variants were introduced on the desired allelic background using either AGT1-Ma or AGT1-Mi transfer vectors. The complete list of *AGXT* variants and associated oligonucleotides used for mutagenesis are reported in Table S1. PCR products were transformed in One Shot Stbl3 *Escherichia coli* chemically competent cells (C7373–03; Thermo Fisher Scientific) using heat shock method (incubations involving 30 min on ice, 45 s at 42 °C, and 2 min on ice). Upon transformation, cells were cultured in 500 μl of SOC medium for 1 h at 37 °C, and then plated on Luria-Bertani agar plates containing ampicillin (100 μg/ml). Mutagenesis was confirmed in isolated plasmids by Sanger sequencing using the primers reported in Table S1. Correctly mutagenized vectors were amplified and extracted using the ZymoPURE II Plasmid Midiprep Kit (Zymo Research Corporation) and used for lentivirus production.

### Lentiviral vector preparation and cell transduction

Lentiviral particles carrying *AGXT* variants were prepared using a second-generation lentiviral system, with cells from the HEK293FT cell line used as packaging cells. HEK293FT cells were transfected transiently using PolyJet reagent (SignaGen Laboratories), according to the manufacturer’s instructions. Briefly, HEK293FT cells (2 × 10^6^) were seeded in 100 mm tissue culture–treated plates (Sarstedt AG & Co KG) and incubated in complete medium for 24 h at 37 °C and with 5% CO_2_. One hour prior to transfection, cells were incubated in complete medium without geneticin and were then transfected with a mixture composed of 1.5 μg of transfer plasmid, 2.25 μg of pCMV-VSV-G envelope plasmid, and 2.25 μg of pCMVR8.74 packaging plasmid, diluted in 250 μl of DMEM medium base. Cells were then mixed with 18 μl of transfection reagent dissolved in 250 μl of DMEM medium base. Transfection mixtures (final volume of 500 μl/reaction) were incubated for 15 min at room temperature (RT), then added dropwise onto cells. After 5 h, the medium was removed, and 6 ml of fresh complete medium (without geneticin) was added for 72 h to each plate. Next, cell media were harvested and centrifuged for 5 min at 800*g*, and supernatants were filtered through 0.45 μm filters (SLGP033RS; Merck Life Science). Finally, lentiviruses were aliquoted and stored at −80 °C.

For cell transduction, *AGXT*-KO HepG2 cells were seeded in complete DMEM in 6-well plates (0.4 × 10^6^/well) 24 h before transduction. Cells were transduced for 24 h in 1 ml of fresh complete medium, added with different amounts of lentiviral preparations (50–500 μl), and in the presence of 12 μg/ml of polybrene (TR-1003-G; Merck Life Science). After transduction, cells were incubated with fresh culture medium for an additional 24 h. Finally, cells were selected using 2.5 μg/ml puromycin (Thermo Fisher Scientific) for 72 h to obtain clones expressing different *AGXT* variants. Afterwards, cells were maintained in standard complete medium.

### Semi-quantitative PCR

To determine the efficiency of viral DNA integration into cellular DNA, genomic DNA was extracted from transduced cells using the Quick-DNA Miniprep Kit (Zymo Research Corporation) and semi-quantitative PCR was performed using primers specific for the amplification of exogenous *AGXT* (*i.e*., h*AGXT*eng) only. Primers for human *GAPDH* were used as an endogenous loading control. PCR protocol was performed as previously described (66), with slight modifications as follows: PCR mixtures consisted of 40 ng of genomic DNA, 0.2 μM primers, and 1× JumpStart REDTaq ReadyMix (1×; Merck Life Science srl), in a final volume of 20 μl/reaction. The PCR thermal profile was 3 min at 95 °C, then 25 cycles of 30 s at 95 °C, 30 s at 60 °C, and 30 s at 72 °C, with a final extension step of 5 min at 72 °C. PCR products were loaded on 1.5% agarose gel stained with SYBR Safe DNA Gel Stain (Thermo Fisher Scientific) and visualized using an iBright CL1500 Imaging System (Thermo Fisher Scientific). Primers sequences used for semi-quantitative PCR were: h*AGXT*eng_F 5′ GGGCTGCGATGATCAACCGA 3′; h*AGXT*eng_R 5′ CAATCGTACCCCGCTGGCAC 3′; h*GAPDH*_F 5′ TGGTATCGTGGAAGGACTCATGAC 3′; h*GAPDH*_R 5′ ATGCCAGTGAGCTTCCGCTTCAGC 3′.

### *AGXT* transaminase activity assay and immunoblot analysis

Cells (4 × 10^6^) were grown in Petri dishes (10 cm) for 24 h and then recovered by cell scraping on ice and pelleted by centrifugation for 5 min at 800*g*. Cell pellets were suspended in 0.1 ml of PBS containing cOmplete protease inhibitor cocktail (1×, Roche Molecular Systems, Inc), and 100 μM PLP, and then lysed by sonication on ice with pulses of 5 s, twice. Cell lysates were centrifuged for 10 min at 13,000*g* at 4 °C, and total protein concentration was measured on recovered supernatants by a standard Bradford assay (B6916, Merck Life Science srl) performed on microplates following the manufacturer's protocol. Total cell lysates obtained from cells expressing *AGXT* variants were used to determine specific AGT enzymatic activity by a standard spectrophotometric assay (29, 67). A fixed amount of total protein (100 μg) was incubated in 100 mM potassium phosphate buffer (pH 7.4) in the presence of 200 μM PLP, 0.5 M L-alanine, and 10 mM glyoxylate, in a final volume of 50 μl, for 30 min at 37 °C. The reactions were stopped by adding 6 μl of trichloroacetic acid (TCA, 10% v/v), and pyruvate production was measured by monitoring NADH oxidation at 340 nm using a spectrophotometric assay coupled with LDH assay.

Cell protein content was measured by Bradford assay in a 96-multiwell plate format with Multiskan SkyHigh spectrophotometer (Thermo Fisher Scientific). For immunoblot analyses, samples (30 μg/lane) were loaded onto sodium dodecyl sulfate-polyacrylamide gels and blotted onto a nitrocellulose membrane through a Trans-Blot Turbo Transfer System (Bio-Rad Laboratories). Membranes were incubated overnight at 4°C with the following primary antibodies: anti-AGT1 (1:10,000) and anti-GO (1:500). As a loading control, membranes were incubated with anti–β-tubulin (1:5000). Filters were then incubated with the appropriate HRP-conjugated secondary antibodies (1:5000) for 1 h. The chemiluminescence signal was detected by an iBright CL1500 Imaging System (Thermo Fisher Scientific). Densitometric analysis was performed using the iBright analysis software (Thermo Fisher Scientific).

### Immunofluorescence

Cells (8 × 10^4^/well) expressing *AGXT* variants were seeded in 24-well plates on polylysine-coated 12 mm coverslips (Knittel glass) and incubated for 24 h. For staining mitochondria, an incubation step of 30 min at 37°C with MitoTracker Red CMXRos (M7512; Thermo Fisher Scientific) was performed prior to fixation with 4% paraformaldehyde dissolved in phosphate-buffered saline (PBS). Next, cells were permeabilized and blocked (40 min at RT with PBS + 3% bovine serum albumin, and 0.3% Triton X-100), and then incubated with the primary antibodies anti-AGT1 (1:5000) and anti–peroxisomal catalase (1:250). After the incubation, samples were washed three times with PBS for 5 min and then incubated with anti-rabbit Alexa Fluor 488 (1:500, A-11008; Thermo Fisher Scientific) or anti–guinea pig Alexa Fluor 555 (1:500, A-21435; Thermo Fisher Scientific) secondary antibodies for 1 h at RT, rinsed in PBS for 5 min three times, counterstained with 10 μg/ml of Hoechst 33,342 (H3570; Thermo Fisher Scientific) for 10 min in PBS, and washed in PBS for 5 min three times before mounting with Dako mounting medium (Agilent Technologies). Image acquisition was performed by a semi-confocal fluorescence microscope equipped with an AxioVision MRm digital camera and ApoTome filtering device (Carl Zeiss) at 63× magnification. 10 different fields were acquired for each variant, consisting of approximately 50 cells per sample (68, 69). Figure cropping and labelling were performed using the GIMP software (ver. 2.10.36).

### Oxalate determination

Oxalate levels were determined in media of cells transfected for 24 h with pCDNA3.1 vector containing human GO cDNA using GenJet reagent (SignaGen Laboratories) (29), and treated for an additional 24 h with or without glycolate challenge (10 mM). After incubation, cell medium was collected and immediately processed. Samples (0.8 ml) were concentrated for 3 h at 37 °C with a SpeedVac Vacuum Concentrator (Thermo Fisher Scientific) until a volume of 100 μl was reached. Oxalate concentration was assessed with a kit provided by Trinity Biotech Plc. following manufacturer’s instructions, with modifications: concentrated samples (100 μl) were diluted with an equal volume of sample diluent reagent and incubated for 5 min at RT with activated charcoal. Samples were then centrifuged for 10 min at 4000×*g* and supernatants (50 μl) were transferred to a new tube containing 250 μl of reagent A. After the addition of 25 μl of reagent B, samples were incubated for 5 min at RT and oxalate concentration was determined by measuring absorbance at 590 nm in a Multiskan SkyHigh microplate reader (Thermo Fisher Scientific).

### Statistical analysis

For data analysis and plotting, GraphPad v.[8.4.2] (Dotmatics, Boston, MA) and R version 4.1.3 https://www.R-project.org/. Accessed May 2024 were used. Data are expressed as mean ± standard deviation (SD). Student’s *t* test was used to assess the significance of differences between two groups, while two-way analysis of variance (ANOVA) followed by Dunnett’s multiple comparison *post hoc* test was used for comparisons including multiple groups and different types of variables. A *p* value < 0.05 was considered statistically significant.

PCA and clustering were performed to group variants based on residual specific activity, protein levels, glyoxylate detoxification ability, and subcellular localization. Glyoxylate detoxification was calculated as the relative reduction of oxalate in the medium as compared with mock LV cells, by setting the reduction of AGT1-Ma cells to 1. For localization, a value of 1 was assigned to variants with peroxisomal localization, 0.5 for mixed mitochondrial/peroxisomal localization, and 0.0 for aberrant mitochondrial localization. The PCA plot was generated with factoextra R package. Data were clustered using hierarchical clustering, while gap statistics embedded in the factoextra package were used to *a priori* determine the optimal number of clusters to be included in the PCA analysis (70), https://CRAN.R-project.org/package=factoextra, Accessed May 2024.

## Data availability

The data sets for the research presented here are available from the corresponding author on reasonable request.

## Supporting information

This article contains supporting information.

## Conflict of interests

The authors declare the following financial interests/personal relationships which may be considered as potential competing interests:

BC: Received grants from Dicerna Pharmaceuticals, Inc.

LG: Nothing to disclose.

IB: Nothing to disclosure.

GR: Consultant for Novo Nordisk.

MG: Former employee of Novo Nordisk.
