# Supplementary material for: Functional analysis of amino acid substitutions within human AGT1 in a cell-based platform to support the diagnosis of primary hyperoxaluria type 1
Source: J Biol Chem. 2025 Jul 17;301(8):110494. doi: 10.1016/j.jbc.2025.110494 (PMC12363580; doi:10.1016/j.jbc.2025.110494)
Supplement: Supplementary data [file mmc1.docx]

**Supplementary Appendix**

This appendix has been provided by the authors to give readers additional information about their work.

**Supplementary Results**

**Table S1.** *AGXT* variant list and oligonucleotides used for site directed mutagenesis and sequencing.

**Fig. S1.** Transduction efficiency of *AGXT* transgene in *AGXT*-KO HepG2 cell clones stably expressing alanine:glyoxylate aminotransferase (AGT1) polymorphic forms.

**Fig. S2.** Infection levels and specificity of transduced *AGXT* variants of unknown significance (VUS) related to alanine:glyoxylate aminotransferase (AGT1) major (AGT1-Ma) polymorphic form.

**Fig. S3.** Infection levels and specificity of transduced catalytically defective *AGXT* variants related to alanine:glyoxylate aminotransferase (AGT1) major (AGT1-Ma) polymorphic form.

**Fig. S4.** Infection levels and specificity of transduced *AGXT* variants of unknown significance (VUS) associated with AGT1 minor (AGT1-Mi) polymorphic form.

**Fig. S5.** Glycolate oxidase overexpression increases oxalate release in cell medium.

**Table S1**

*AGXT* variant list and oligonucleotides used for site-directed mutagenesis and sequencing.

| **Mutagenesis oligonucleotides**** | | | |  |
| --- | --- | --- | --- | --- |
| ***AGXT* allele** | **Amino acid substitution** | | **Primer sequences (5’-3’)** | |
| AGT1-Ma | p.Pro11Leu | | F: acttctcgtcactccgc**t**taaagctttgctgaaac  R: gtttcagcaaagcttta**a**gcggagtgacgagaagt | |
| AGT1-Ma | p.Pro28Ser | | F: CCAAATCAACTGTTGCTTGGC**T**CAGGGCCG  R: CGGCCCTG**A**GCCAAGCAACAGTTGATTTGG | |
| AGT1-Ma | p.Gly41Arg | | F: ccaggattatggctgct**c**gtggtttgcaaatgatc  R: gatcatttgcaaaccac**g**agcagccataatcctgg | |
| AGT1-Ma | p.Arg118Cys | | F: cagttgatattggcgaa**t**g**c**attggcgctagggtcca  R: tggaccctagcgccaat**g**c**a**ttcgccaatatcaactg | |
| AGT1-Ma | p.Asp129His | | F: gtccatcccatgactaaa**c**atcccggcgg  R: ccgccgggat**g**tttagtcatgggatggac | |
| AGT1-Mi | p.Phe152Ile | | F: cacaacataaacccgttctcctt**a**tcctcacacatg  R: catgtgtgagga**t**aaggagaacgggtttatgttgtg | |
| AGT1-Ma | p.Ser158Leu | | F: TTCTCCTTTTCCTCACACATGGTGAA**CT**TAGCACGGGAGTTC  R: gaactcccgtgcta**ag**ttcaccatgtgtgaggaaaaggagaa | |
| AGT1-Mi | p.Gly170Arg | | F: TCTCGACGGATTT**C**GCGAGCTGTGTCATCG  R: CGATGACACAGCTCGC**G**AAATCCGTCGAGA | |
| AGT1-Ma | p.Asp183Asn | | F: tataaatgtttactgttagtc**a**actcagttgccagtttaggcg  R: cgcctaaactggcaactgag**t**tgactaacagtaaacatttata | |
| AGT1-Ma | p.Ala186Val | | F: gttagtcgactcagttg**t**cagtttaggcggcactc  R: gagtgccgcctaaactg**a**caactgagtcgactaac | |
| AGT1-Ma | p.Arg197Gln | | F: ggcactcctctgtatatggatc**aa**cagggaattgatattctctatt  R: aatagagaatatcaattccctg**tt**gatccatatacagaggagtgcc | |
| AGT1-Ma | p.Ala248Val | | F: agatattaaatggctcg**t**gaacttttggggctgcg  R: cgcagccccaaaagttc**a**cgagccatttaatatct | |
| AGT1-Mi | p.Glu274Asp | | F: ctctatagtctccggga**c**tctctcgctctgatc  R: gatcagagcgagaga**g**tcccggagactatagag | |
| AGT1-Mi | p.Ile279Thr | | F: cgggaatctctcgctctga**c**cgctgagcaa  R: ttgctcagcg**g**tcagagcgagagattcccg | |
| AGT1-Mi | p.Arg289Cys | | F: aggtttggaaaattcctgg**t**g**t**caacatagagaagctgctg  R: cagcagcttctctatgttg**a**c**a**ccaggaattttccaaacct | |
| AGT1-Ma | p.Arg289His | | F: GAGCAAGGTTTGGAAAATTCCTGGC**AT**CAACATAGAGAAG  R: CTTCTCTATGTTG**AT**GCCAGGAATTTTCCAAACCTTGCTC | |
| AGT1-Ma | p.Pro314Pro | | F: ttgtttgtcaaagatcc**a**gccttaaggctccctac  R: gtagggagccttaaggc**t**ggatctttgacaaacaa | |
| AGT1-Ma | p.Arg317Trp | | F: tgtttgtcaaagatcctgcctta**t**ggctccctactg  R: cagtagggagcc**a**taaggcaggatctttgacaaaca | |
| AGT1-Ma* | p.Ile340Met | | F: gcgggatattgtttcctatgtgat**g**gatcattttgatatagaaattatgg  R: ccataatttctatatcaaaatgatc**c**atcacataggaaacaatatcccgc | |
| AGT1-Ma | p.Gly365Cys | | F: aagtacttagaataggattactc**t**gttgtaacgctactagagaaaac  R: gttttctctagtagcgttacaac**a**gagtaatcctattctaagtactt | |
| **Sequencing oligonucleotides** | | | |  |
| CMV_F | | CGCAAATGGGCGGTAGGCGTG | |  |
| h*AGXT*eng*_*Seq1_F** | | TAAACTTCTCGTCACTCCGCC | |  |
| h*AGXT*eng*_*Seq2_F** | | GCGGCACTCCTCTGTATATG | |  |
| *AGT1-Ma + p.Pro11Leu; **Mutagenesis and sequencing primers were designed to be complementary to codon optimized version of *AGXT* cDNA; Bold and underlined nucleotides indicate base and codon changes, respectively. | | | |  |


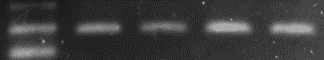


*AGXT*

*GAPDH*

200

300

100

200

bp

AGT1-Ma 50

WT

mock LV

AGT1-Mi 100


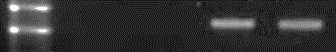


**Fig. S1.** Transduction efficiency of *AGXT* transgene in *AGXT*-KO HepG2 cell clones stably expressing alanine:glyoxylate aminotransferase (AGT1) polymorphic forms.

Genomic DNA was extracted from *AGXT*-KO HepG2 cells transduced with 50 (AGT1-Ma 50) or 100 µl (AGT1-Mi 100) of lentiviral vectors containing medium, encoding for AGT1 major (AGT1-Ma) or AGT1 minor (AGT1-Mi), respectively. Semi-quantitative polymerase chain reaction (PCR) analysis was performed using specific primers for transgene amplification only, as well as for *GAPDH* as endogenous genome reference. PCR products were analyzed by agarose gel electrophoresis. bp, base pairs; LV, lentivirus; WT, wild type.

35 kDa

Anti-Pro11

Anti-Leu11

β-tubulin

AGT1-Ma

p.Pro28Ser-Ma

mock LV

p.Gly41Arg-Ma

p.Arg118Cys-Ma

p.Asp129His-Ma

p.Ala186Val-Ma

p.Arg197Gln-Ma

p.Ala248Val-Ma

p.Arg289His-Ma

p.Pro314Pro-Ma

p.Arg317trp-Ma

p.Gly365Cys-Ma


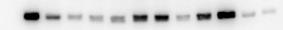

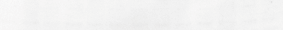


35 kDa

55 kDa


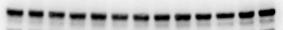


B

A

*AGXT*

*GAPDH*

200

300

100

200

bp


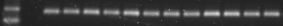

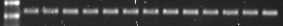


AGT1-Ma

p.Pro28Ser-Ma

mock LV

p.Gly41Arg-Ma

p.Arg118Cys-Ma

p.Asp129His-Ma

p.Ala186Val-Ma

p.Arg197Gln-Ma

p.Ala248Val-Ma

p.Arg289His-Ma

p.Pro314Pro-Ma

p.Arg317trp-Ma

p.Gly365Cys-Ma

**Fig. S2.** Infection levels and specificity of transduced *AGXT* variants of unknown significance (VUS) related to alanine:glyoxylate aminotransferase major allele (AGT1-Ma).

(A) Amplification of genomic DNA extracted from *AGXT*-KO HepG2 cells expressing *AGXT* VUS associated with AGT1-Ma by semi-quantitative polymerase chain reaction (PCR). *GAPDH* was used as loading control. (B) Cell lysates were immunoblotted with AGT1 polymorphic forms-selective anti-Pro11 (AGT1-Ma) and anti-Leu11 (AGT1 minor [AGT1-Mi]) antibodies. β-tubulin was used as loading control. bp, base pairs; LV, lentivirus.


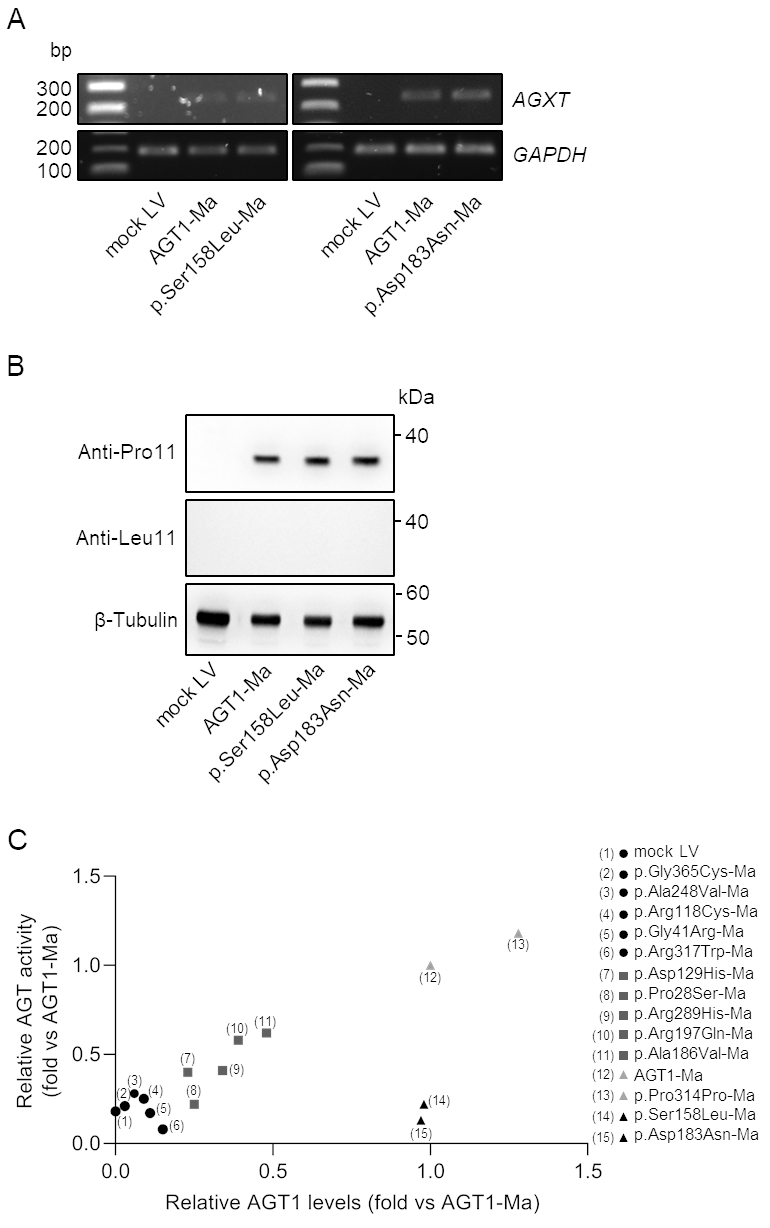


**Fig. S3.** Infection levels and specificity of transduced *AGXT* catalytically defective variants related to alanine:glyoxylate aminotransferase major allele (AGT1-Ma).

(A) Amplification of genomic DNA extracted from *AGXT*-KO HepG2 cells expressing *AGXT* catalytically defective variants associated with AGT1-Ma by semi-quantitative polymerase chain reaction (PCR). *GAPDH* was used as loading control. (B) Cell lysates were immunoblotted with AGT1 polymorphic forms-selective anti-Pro11 (AGT1-Ma) and anti-Leu11 (AGT1 minor [AGT1-Mi]) antibodies. β-tubulin was used as loading control. (C) Correlation between transaminase specific activity and AGT1 protein levels in clones expressing *AGXT* VUS associated to AGT1-Ma. Clusters represent *AGXT* variants with strong (black circle), moderate (grey square), or absent (grey triangle) reduction in enzyme activity and protein levels, and catalytically defective *AGXT* variants (black triangle) with impaired enzyme activity and unaltered protein levels. bp, base pairs; LV, lentivirus.

**
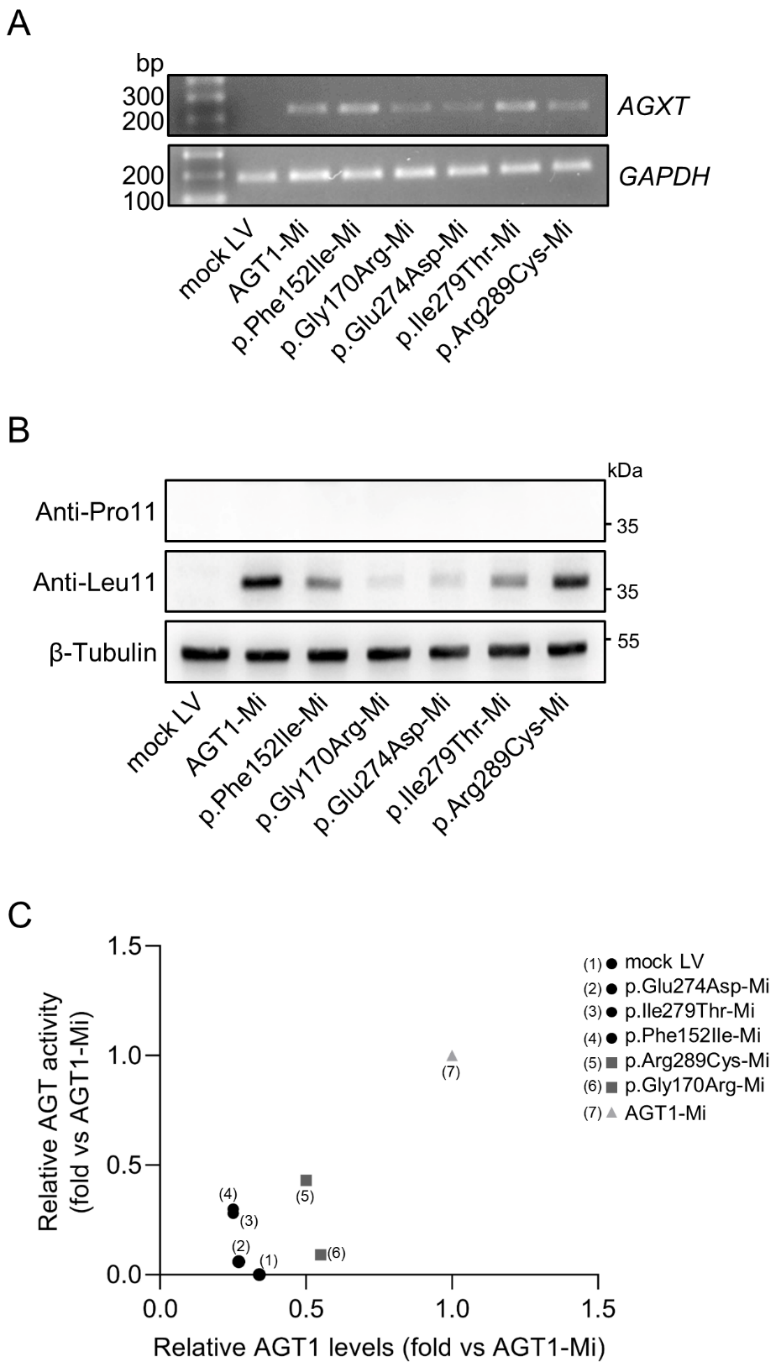
**

**Fig. S4.** Infection levels and specificity of transduced *AGXT* variants of unknown significance (VUS) associated with AGT1 minor allele (AGT1-Mi).

(A) Amplification of genomic DNA extracted from *AGXT*-KO HepG2 cells expressing *AGXT* VUS associated with AGT1-Mi by semi-quantitative PCR. *GAPDH* was used as loading control. (B) Cell lysates were immunoblotted with alanine:glyoxylate aminotransferase (AGT1) polymorphic forms-selective anti-Pro11 (AGT1-Ma]) and anti-Leu11 (AGT1-Mi) antibodies. β-tubulin was used as loading control. (C) Correlation between transaminase specific activity and AGT1 protein levels in clones expressing *AGXT* VUS associated to AGT1-Mi. Clusters represent *AGXT* variants with strong (black circle), moderate (grey square), or absent (grey triangle) reduction in enzyme activity and protein levels. bp, base pairs; LV, lentivirus.


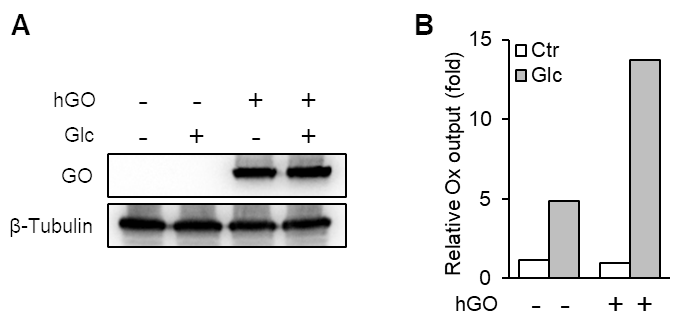


**Fig. S5.** Glycolate oxidase overexpression increases oxalate release in cell medium.

*AGXT*-KO HepG2 cells (mock lentivirus [LV]) were transfected with pCDNA3.1 vector encoding for human glycolate oxidase (GO) and then, after 24 h, treated or not treated (Ctr, untreated cells) with 10 mM glycolate (Glc) for 24 h. After treatment, cells were analyzed (A) for GO expression by immunoblot and media were recovered for (B) oxalate concentration measurements. β-tubulin was used as loading control. Ctr, control (untreated cells); hGO, human glycolate oxidase; Glc, glycolate; Ox, oxalate
